# Assessment of prescribing errors reported by community pharmacy professionals

**DOI:** 10.1186/s40545-022-00461-9

**Published:** 2022-10-15

**Authors:** Wudneh Simegn, Berhanemeskel Weldegerima, Mohammed Seid, Ayal Zewdie, Dawit Wondimsigegn, Chilot Abyu, Asmamaw Emagn Kasahun, Abdulwase Mohammed Seid, Gashaw Sisay, Yigizie Yeshaw

**Affiliations:** 1 Department of Social and Administrative Pharmacy, School of Pharmacy, University of Gondar, P.O. Box 196, Gondar, Ethiopia; 2 Gondar University Referral Hospital, University of Gondar, P.O. Box 196, Gondar, Ethiopia; 3 Department of Pharmaceutics, School of Pharmacy, University of Gondar, P.O. Box 196, Gondar, Ethiopia; 4 Department of Clinical Pharmacy, School of Pharmacy, University of Gondar, P.O. Box 196, Gondar, Ethiopia; 5 Department of Physiology, School of Medicine, University of Gondar, P.O. Box 196, Gondar, Ethiopia

**Keywords:** Prescribing errors, Community pharmacists, Ethiopia

## Abstract

**Background:**

Medication errors have serious consequences for patients’ morbidity and mortality. The involvement of pharmacy professionals in the prescribing and dispensing procedure allowed the detection of a range of drug-related problems in addition to identification by prescribers. They are often the first point of contact in the healthcare system in identifying prescribing errors and intervening in these errors by dealing with the prescribers and the patients.

**Objectives:**

This study aimed to assess prescribing errors reported by community pharmacy professionals in Gondar Town, North West Ethiopia.

**Methods:**

A self-administered cross-sectional survey was employed from February 29 to June 23, 2020, to collect data on prescribing errors reported by community pharmacy professionals. All community pharmacy professionals found in Gondar town were included. Community pharmacy professionals who were ill at the time of study and who had less than 6 months of work experience were excluded.

**Results:**

Seventy-four pharmacy professionals participated in the study with a response rate of 93.6%. The overall prevalence of prescribing errors was 75.1% (95% CI 71.08–78.70). Of these errors, drug selection was the most common (82.4%), followed by errors of commission (79.7%) and errors of omission (78.4%). Antibiotics (63.5%) were commonly involved in prescribing errors, followed by analgesics (44.5%) and antipsychotics (39.5%).

**Conclusion:**

The findings of this study revealed a high prevalence of prescribing errors in Gondar, Ethiopia. Drug selection was the most prescribed error, followed by errors of commission. Stakeholders should design interventions such as training, integrating prescribers with clinical pharmacists and supervising interns by seniors. Large-scale studies that include potential factors of prescribing problems are recommended for future researchers.

## Background

The prevention of medication errors should be prioritized in all health care systems in the world [1, 2] as drugs are the most important and cost-effective elements of health care that help to cure diseases and relieve symptoms [3, 4]. Prescription errors were defined as any error identified in the process of dispensing that might interfere with the dispensing of prescriptions, such as incomplete prescriptions and prescriptions with incorrect information, which is one of the causes of treatment failure that leads to frequent, serious, and expected events in critical care units [1, 5, 6] to result in patient morbidity and mortality [7]. Prescribing errors can occur at the stage of medication prescribing by the prescribers, those who label, package, compound, dispense and others like absence of education or monitoring during the use of the medicine [8].

Pharmacists are health professionals that dispense different medications to improve patients’ health outcomes and provide competency-based general practice in the health care system [9–12]. They play an active role in preventing and solving drug-related problems for the members of society who contact them by prescription or for OTC drug requirements [2, 13]. Community pharmacies are often the first point of contact in the healthcare system [14, 15]. The major activities of community pharmacists are: the processing of prescriptions given (by piece of paper, email, phone call, or other means from prescribers who have directly contacted the actual patients), giving care to patients; or providing clinical pharmacy services [16]. The involvement of pharmacists in the prescribing and dispensing procedures allowed detection of a range of drug-related problems [17].

Currently, there are more than 700 community pharmacists in Ethiopia. Community pharmacists are consistently assessing prescriptions for potential mistakes, including prescribing errors, before the drugs are dispensed. They identify, record, rectify, and reduce the occurrence of prescribing errors (errors of omission, errors of commission, and so on) [18]. Community pharmacists try to give suggestions and open discussion to correct clinical problems or to provide their patients with more reasonable therapy [19]. Suggested interventions by clinical pharmacists to prevent drug-related problems are mostly accepted and implemented by the prescribers [20]. In most drug delivery systems, the pharmacist represents the final point at which prescribing errors and related problems can be identified and corrected without threatening the quality of care that is delivered to the patient [13]. The inter-professional relationship should be highly consolidated and maintained in the delivery of pharmaceutical care in the community setting [21]. As a result, screening prescription orders for problems and intervening to correct drug prescription problems that are identified by the pharmacist could be recognized as a central component of the pharmacist’s responsibilities to the patient or the customer [4, 13].

Though inappropriate, ineffective, and economically inefficient use of drugs are common problems worldwide, they are particularly pronounced in low-income countries [3]. Health system administrators require data about the pattern of drug use, specific problems in drug use, and ways of monitoring drug use over a period of time [22]. Appropriate use of drugs in the health care system can be important not only for financial reasons, but also for the concern of policy-makers and managers to improve the health care system [3]. Therefore, this study was aimed at assessing prescribing errors reported by community pharmacy professionals in Gondar, Ethiopia.

## Methods

### Study setting, design, and period

A cross-sectional study was conducted from February 29 to June 23, 2019 in Gondar town community pharmacies. Gondar town is located 728 km away from Addis Ababa, the capital city of Ethiopia. According to the 2007 population and housing census report, Gondar town has an estimated population of 206,987. The report of the Gondar town health administration office reveals that the town has 19 community pharmacies and 33 drug stores.

### Study population

All pharmacy professionals working in Gondar town community pharmacy who were present at the time of the data collection period were included in the study population. Those pharmacy professionals who were severely ill during the data collection period and had work experience of less than 6 months were excluded.

### Sample size and data collection procedure

To get the maximum sample size, all pharmacists (bachelor of pharmacy) and druggists (diploma in pharmacy) who worked in community pharmacies in Gondar town were included. A structured, pretested, self-administered questionnaire consisting of sociodemographic characteristics and common prescribing errors was used to collect the required data for the study. The questions were adapted from various sources [1, 13]. Two pharmacy technicians participated in distributing and returning the questionnaire.

### Variables of the study

In this study, the dependent variable was prescribing error, and the independent variables were sex, age in years, marital status, educational level, site of work, work experience, and number of customers’ visits/day.

### Statistical analysis and data quality control

The data were entered into Epi-info 7.1 and exported to SPSS version 20 for further statistical analysis. The range, mean with standard deviation (SD), frequency, and percent were computed to articulate the descriptive results of the study. To assure the data quality, high emphasis was given to the data collection instrument. The questionnaire was pretested, and data collection facilitators were trained about the purpose of the study and ethical issues 2 days before the actual data collection began.

## Results

### Socio-demographic characteristics

Seventy-four pharmacy professionals participated in the study with a response rate of 93.6%. The majority of the respondents were male (64.9%), aged 20–30 years of age (67.6%), and about half of the respondents (51.4%) were single. About 64.9% of respondents were pharmacists (bachelor of pharmacy) and the remaining were druggists (diploma) holders (Table 1).

**Table 1:** Socio-demographic characteristics of respondents (*N* 74)

Characteristics	Frequency (*n*)	Percent (%)
Gender		
Male	48	64.9
Female	26	35.1
Age (years)		
20–30	50	67.6
31–40	17	23
41–50	4	5.4
> 50	3	4
Marital status		
Single	38	51.4
Married	36	48.6
Educational status		
Druggist	25	33.8
Pharmacist	49	66.2
Site of work		
Public community pharmacy	27	36.5
Private community pharmacy	43	58.1
Red cross community pharmacy	4	5.4
Work experience (years)		
1–5	35	47.3
> 5	39	52.7
Number of customers visit/day		
< 50	30	40.5
51–100	17	23
101–200	16	21.6
> 200	11	14.9

### Prevalence of prescribing errors

The overall prevalence of prescribing errors in this study was 75.1% [95% CI (71.08–78.70)]. Drug selection was the common prescribing problem (82.4%), followed by errors of commission (79.7%) (Table 2). Sixty-six (89.2%) of community pharmacists reported that there was no difference in the frequency of prescribing errors among male and female patients. More than half of the community pharmacists responded that adults (56.8%) and children (47.3%) had faced common prescribing errors. According to the findings of this study, 56.8% of prescribing errors were made by interns (Table 3).

**Table 2:** Frequency distribution of prescribing errors encountered in the study (*N* = 74)

Variables	Responses	
Types of prescribing problems	Yes *n* (%)	No *n* (%)
Errors of omission	58 (78.4)	16 (21.6)
Incomplete or unavailable form/strength	53 (71.6)	21 (28.4)
Violates legal requirements	40 (54.1)	34 (45.9)
Quantity/duration not specified	34 (45.9)	40 (54.1)
Dose/regimen not specified	31 (41.9)	43 (58.1)
Illegible	29 (39.2)	45 (60.8)
Errors of commission	59 (79.7)	15 (20.3)
Incorrect regimen	53 (71.6)	21 (28.4)
Duplicate therapy	40 (54.1)	34 (45.9)
Incorrect drug/indication	39 (52.7)	35 (47.3)
Incorrect form	39 (52.7)	35 (47.3)
Incorrect quantity/duration	39 (52.7)	35 (47.3)
Incorrect patient	22 (29.7)	52 (70.3)
Drug selection	61 (82.4)	13 (17.6)
Is the prescriber use inappropriate drug?	48 (64.9)	26 (35.1)
More cost-effective drug available?	40 (54.1)	34 (45.9)
Is the indication of the drug mentioned?	39 (52.7)	35 (47.3)
Inappropriate drug form?	39 (52.7)	35 (47.3)
Synergistic/preventive drug required and not given?	37 (50.0)	37 (50.0)
Is there Inappropriate combination of drugs?	34 (45.9)	40 (54.1)
Inappropriate duplication of therapeutic group or active ingredient?	28 (37.8)	46 (62.2)
No alternative?	17 (23.0)	57 (77.0)
Dose selection and treatment duration	56 (75.7)	18 (24.3)
Dosage regimens not frequent enough?	41 (55.4)	33 (44.6)
Deterioration/improvement of disease state requiring dose adjustment?	40 (54.1)	34 (45.9)
Duration of treatment too short?	38 (51.4)	36 (48.6)
Drug dose too low?	37 (50.0)	37 (50.0)
Drug dose too high?	36 (48.6)	38 (51.4)
Dosage regimen too frequent?	32 (43.2)	42 (56.8)
Duration of treatment too long?	29 (39.2)	45 (60.8)
Drug interaction	44 (59.5)	30 (40.5)
Drug–drug interaction	43 (58.1)	31 (41.9)
Hypersensitivity reaction	21 (28.4)	53 (71.6)

**Table 3:** Patient and prescriber characteristics (*N* = 74)

Variables	Responses	
	Frequency *n*	Percent (%)
Patient gender encountering prescribing errors		
Both	66	(89.2)
Male	4	(5.4)
Female	4	(5.4)

	Yes *n* (%)	No *n* (%)
Patient age (in years)		
Adult (19–65)	42 (56.8)	32 (43.2)
Child (< 13)	35 (47.3)	39 (52.7)
Elderly (> 65)	27 (36.5)	47 (67.5)
Adolescent (13–18)	19 (25.7)	55 (74.3)

	Yes *n* (%)	No *n* (%)
Prescriber performing prescribing errors		
Interns	42 (56.8)	32 (43.2)
General practitioners	31 (41.9)	43 (58.1)
Specialists	23 (31.1)	51 (68.9)
Nurses	22 (29.7)	52 (70.3)
Health officer	13 (17.6)	61 (82.4)
Anesthetics	10 (13.5)	54 (86.5)

**Fig. 1:**
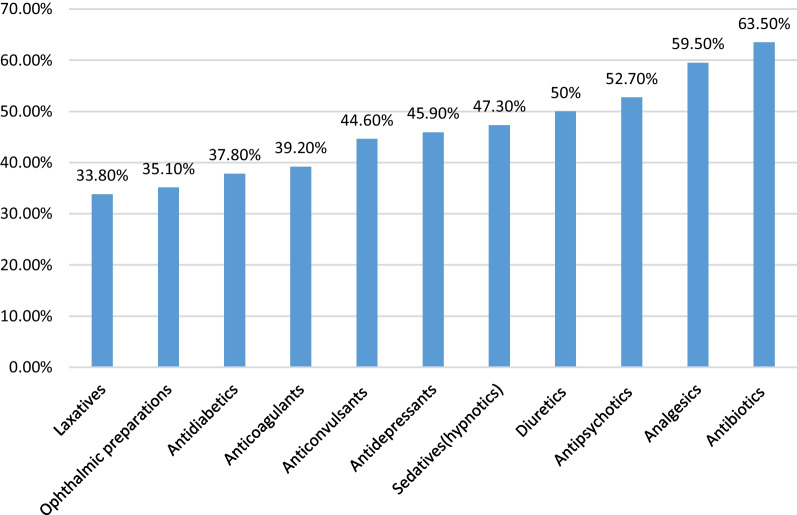
Common classes of drugs involved as problematic prescription orders (*N* = 74)

In this study, about 11 classes of drugs were reported as problematic prescription orders. Antibiotics (63.5%) were the major class of drugs commonly involved in problematic prescription orders, followed by analgesics (59.5%) (Fig. 1).

## Discussion

Community pharmacists are health professionals who analyze prescriptions during the initial stage of dispensing, allowing the identification of a variety of drug-related problems and serving as the primary source of patient information [13, 17]. This is the first study to assess the prevalence of prescribing errors among community pharmacy professionals in Ethiopia.

In this study, the overall prevalence of prescribing errors was 75.1% (95% CI 71.08–78.70). The high prevalence of prescribing errors in the current study might be due to the lack of drug knowledge of prescribers, lack of attention in patient care, and unavailability of essential drug lists in hard copy at each health facility [14]. The finding of this study is higher compared to other studies in Ethiopia [2, 5, 7, 23]. The higher prevalence of prescribing errors in the current study might be due to the inclusion of errors related to the illegality of prescriptions (illegible handwriting and lack of authentication). However, this finding is lower than other studies conducted elsewhere [1, 13]. This could be related to the difference in study setting, healthcare system, and methodology (Rupp et al. used direct observation, while the current study was collected by a self-administered questionnaire).

In the current study, most of the respondents (82.4%) identified that drug selection was the most common prescribing error, followed by errors of commission (79.7%). It is in line with the study done at Jimma University specialized hospital [7]. But the other studies reported that errors of omission were the major prescribing error [5, 13, 23]. The variation might be due to the source of the study subjects, methodology, or time of the study.

From errors of commission, incorrect dose/regimen was the most identified prescribing error (71.6%), followed by duplicate therapy (54.1%) and incorrect drug/indication (52.7%). The commission assessed that problems consisted of prescription orders that were incorrect or inappropriate [2]. This might have happened due to a lack of knowledge, experience, and negligence of prescribers. From error of omission, incomplete or unavailable forms/strengths were identified as prescribing problems by most respondents (71.6%). The error of omission implies that prescription orders were incomplete about some essential prescribing information [13]. Another study identified it as a major prescribing problem [24]. Such kinds of problems might also happen due to negligence and work load [23].

From dose selection and treatment duration, the most common errors mostly identified were dosage regimens not frequent enough, improvement of disease state requiring dose adjustment, and duration of treatment too short, with 55.4%, 54.1%, and 51.4%, respectively. This could happen as prescribers might not have adequate knowledge, experience, and skills for the patient’s case as well as ignorance to share ideas with seniors and clinical pharmacists.

From drug interaction, the most identified prescribing errors were drug–drug interaction and hypersensitivity reactions, with 58.1% and 28.4%, respectively. As there might be polypharmacy, especially for chronic patients, the possibility of drug–drug interaction could be high [17]. Therefore, in the accessibility of the drug information center, lack of updated knowledge and poor experience of prescribers could lead to prescribing errors having serious effects on patients due to drug interaction.

Interns (56.8%) and general practitioners (41.9%) were the most reported inappropriate prescribers as compared to others. This is because interns have less experience in their work than others [25, 26]. Most respondents reported that antibiotics (63.5%) and analgesics (59.5%) were the major classes of drugs commonly involved in problematic prescriptions. Similar findings were observed in other studies conducted elsewhere [2, 7, 23]. It might be due to the fact that the above listed drugs were the most commonly prescribed drugs for many cases, so the probability of detecting prescribing errors from these drug groups could be high [23].

The study’s limitations included a small sample size (the number of pharmacy professionals in a community pharmacy setting in the town is low) and recall bias. Despite these limitations, the current study will add important information about the status of prescribing errors in the absence of similar literature in the country (the majority of previous studies were not conducted at the community pharmacy level).

## Conclusion

The current study showed that there is a high prevalence of prescribing errors reported by community pharmacies in Gondar, Ethiopia. Drug selection was the most reported error, and interns were highly reported to have made prescribing errors. Antibiotics and analgesics were the most common prescription errors. Adequate training for prescribers, integrating prescribers with clinical pharmacists, and senior supervision of interns would all help to reduce prescribing errors. Large-scale studies that include potential factors of prescribing problems are recommended for future researchers.

## Data Availability

The dataset is accessible at the corresponding author upon reasonable request.
